# Supplementary material for: Impact of Seasonal Consumption of Local Tomatoes on the Metabolism and Absorption of (Poly)Phenols in Fischer Rats
Source: Nutrients. 2022 May 13;14(10):2047. doi: 10.3390/nu14102047 (PMC9144325; doi:10.3390/nu14102047)
Supplement: Supplementary file 1 [file nutrients-14-02047-s001.zip › nutrients-1711945-supplementary.pdf]

Table S1. Proximate composition of local (LT) and non-local (NLT) tomatoes.

Components	LT	NLT
Ash	8.61 ± 0.28	8.76 ± 0.12
Protein	13.54 ± 0.04	12.96 ± 0.00 *
Total carbohydrate, by difference	75.18 ± 0.14	75.74 ± 0.14
Total dietary fiber	25.26 ± 0.00	24.31 ± 1.18
Total sugar	36.98 ± 0.93	39.57 ± 0.92 *
Total lipid (fat)	2.51 ± 0.03	2.49 ± 0.01

The results are expressed as g/100 g dry weight (dw) ± standard deviation (SD) ($n = 3$). * denotes a significant difference ($p < 0.05$) by the Student's t-test. Table adapted from Cruz-Carrión et al. [1]. LT, local tomatoes; NLT, non-local tomatoes.

Table S2. Rat serum metabolites of tomato (poly)phenolic compounds monitored through uHPLC-MS^n.

Metabolite	RT	[M-H]- (*m*/*z*)	MS^2 ions (*m*/*z*)	Standard Compound Used for Quantification
3-(3′-methoxyphenyl)propanoic acid-4′-glucuronide	4.49	371	**175**, 113, 195	3-(3′-methoxyphenyl)propanoic acid-4′-glucuronide
3-(3′-methoxyphenyl)propanoic acid-4′-sulfate	4.60	275	**195**, 149	3-(3′-methoxyphenyl)propanoic acid-4′-sulfate
3-(4′-hydroxyphenyl)propanoic acid-3′-glucuronide	4.33	357	**181**, 175, 113, 137	3-(4′-hydroxyphenyl)propanoic acid-3′-glucuronide
3-(4′-hydroxyphenyl)propanoic acid-3′-sulfate	4.4	261	**181**, 135, 137	3-(4′-hydroxyphenyl)propanoic acid-3′-sulfate
3-(4′-methoxyphenyl)propanoic acid-3′-glucuronide	4.96	371	**175**, 195, 113	3-(4′-methoxyphenyl)propanoic acid-3′-glucuronide
3′-hydroxycinnamic acid-4′-glucuronide	3.68	355	**179**, 175, 135, 113	3′-hydroxycinnamic acid-4′-glucuronide
3′-methoxycinnamic acid-4′-glucuronide	4.12	369	**175**, 193, 113, 178	3′-methoxycinnamic acid-4′-glucuronide
3′-methoxycinnamic acid-4′-sulfate	4.70	273	**193**, 229, 149	3′-methoxycinnamic acid-4′-sulfate
4′-hydroxycinnamic acid-3′-glucuronide	4.37	355	**179**, 175, 135	3′-hydroxycinnamic acid-4′-glucuronide
4′-methoxycinnamic acid-3′-glucuronide	4.89	371	**175**, 113, 193	3′-methoxycinnamic acid-4′-glucuronide
4′-methoxycinnamic acid-3′-sulfate	5.06	273	**193**, 149	3′-methoxycinnamic acid-4′-sulfate
Hydroxycinnamic acid-sulfate I	4.36	259	**179**, 215, 135	3-(4′-hydroxyphenyl)propanoic acid-3′-sulfate
Hydroxycinnamic acid-sulfate II	4.65	259	**179**, 215, 135	3-(4′-hydroxyphenyl)propanoic acid-3′-sulfate
Naringenin-4′-glucuronide	6.74	447	**271**, 175	Quercetin-3-glucuronide
Naringenin-7-glucuronide	6.64	447	**271**, 175	Quercetin-3-glucuronide
Quercetin-3-glucuronide	5.96	477	**301**, 179	Quercetin-3-glucuronide
Quercetin-3′-sulfate	7.46	381	**301**, 179	Quercetin-3′-sulfate

The fragment ions are reported in their order of relative abundance. Quantifier ions are reported in bold. Standard of both naringenin-glucuronides have been only used for identification because of their lower purity than standard of quercetin-3-glucuronide. The limit of detection (LOD) and quantification (LOQ) for all used standards were evaluated. For glucuronide standards (LOD = 0.01 μM, LOQ = 0.05 μM) and for sulfate standards (LOD = 0.001 μM, LOQ = 0.005 μM). uHPLC-MS^n, ultra-high-performance liquid chromatography coupled with mass spectrometry and linear ion trap mass spectrometric; RT, retention time.

Filename: nutrients-1711945-supplementary.docx
Directory: E:\5.12\symmetry-1257029
Template: C:\Users\MDPI\AppData\Roaming\Microsoft\Templates\Normal.dotm
Title: T
Subject:
Author: MDPI
Keywords:
Comments:
Creation Date: 4/20/2022 4:48:00 PM
Change Number: 8
Last Saved On: 5/13/2022 3:53:00 PM
Last Saved By: MDPI 94
Total Editing Time: 13 Minutes
Last Printed On: 5/13/2022 5:41:00 PM
As of Last Complete Printing
Number of Pages: 2
Number of Words: 879 (approx.)
Number of Characters: 4,831 (approx.)
